# Research Advances in Wood Composites in Applications of Industrial Wastewater Purification and Solar-Driven Seawater Desalination

**DOI:** 10.3390/polym15244712

**Published:** 2023-12-14

**Authors:** Dongsheng Song, Dingqiang Zheng, Zhenghui Li, Chengyu Wang, Jian Li, Ming Zhang

**Affiliations:** 1 Key Laboratory of Wooden Materials Science and Engineering of Jilin Province, School of Material Science and Engineering, Beihua University, Jilin 132013, China; sds230217@163.com (D.S.); 13982758633@163.com (D.Z.); lizhenghui202304@163.com (Z.L.); 2 Key Laboratory of Bio-Based Material Science and Technology, Ministry of Education, School of Material Science and Engineering, Northeast Forestry University, Harbin 150040, China; wangcy@nefu.edu.cn (C.W.); nefujianli@163.com (J.L.)

**Keywords:** wood composites, polymers, nano/micropore structure, industrial wastewater purification, solar-driven seawater desalination

## Abstract

In recent years, the ecosystem has been seriously affected by sewage discharge and oil spill accidents. A series of issues (such as the continuous pollution of the ecological environment and the imminent exhaustion of freshwater resources) are becoming more and more unmanageable, resulting in a crisis of water quality and quantity. Therefore, studies on industrial wastewater purification and solar-driven seawater desalination based on wood composites have been widely considered as an important development direction. This paper comprehensively analyzes and summarizes the applications of wood composites in the fields of solar-driven seawater desalination and polluted water purification. In particular, the present situation of industrial wastewater containing heavy metal ions, microorganisms, aromatic dyes and oil stains and related problems of solar-driven seawater desalination are comprehensively analyzed and summarized. Generally, functional nanomaterials are loaded into the wood cell wall, from which lignin and hemicellulose are selectively removed. Alternatively, functional groups are modified on the basis of the molecular structure of the wood microchannels. Due to its three-dimensional (3D) pore structure and low thermal conductivity, wood is an ideal substrate material for industrial wastewater purification and solar-driven seawater desalination. Based on the study of objective conditions such as the preparation process, modification method and selection of photothermal conversion materials, the performances of the wood composites in filtration, adsorption and seawater desalination are analyzed in detail. In addition, this work points out the problems and possible solutions in applying wood composites to industrial wastewater purification and solar-driven seawater desalination.

## 1. Introduction

Wood is composed of various tissue structures, cell morphologies, pore structures and chemical compositions. Therefore, it is a kind of polymer-based natural composite with a hierarchical and porous structure. Meanwhile, it also has obvious anisotropy, from the meter-level trunk to the decimeter- and centimeter-level wood fibers, millimeter-level annual rings and micron-level wood cells. Up to the cellulose nanofibrils, it has an extremely delicate and orderly multi-scale hierarchical structure [1,2].

Wood can be divided into coniferous wood and broadleaf wood. Coniferous wood mainly includes axial tracheids, wood rays, axial parenchyma and resin canals. Broadleaf wood mainly includes conduits, wood fibers, axial parenchyma and wood rays (a few types contain certain tracheids). It is these wood cells with different shapes, sizes and arrangements that form wood through an orderly and close combination. Then, they can create wood’s unique pore structure [3]. According to their size, voids in wood can be divided into macropores, micropores and mesopores: (1) Macropores refer to pores that can be seen by the naked eye. Examples include wood cells (width: 50~1500 μm; length: 0.1~10 mm), vessels (20~400 μm), tracheids (15~40 μm) and intercellular canals (50~300 μm). (2) A micropore is a void with the order of magnitude of a molecular chain cross-section as the maximum starting point. For instance, the cross-section of the cellulose molecular chain is of the order of magnitude of a micropore. (3) Mesopores refer to voids with one, two or three dimensions in the nanometer scale (1~100 nm). For instance, there are marginal pores (10 nm~8 μm), simple pit pores (50~300 nm) and wood cell wall gaps (2~10 nm) in a dry or wet state and microfibril gaps (1~10 nm) in a swollen state in coniferous wood [4].

The wood cell wall is made up of approximately 45% cellulose (linear polymer composed of β-D-glucose) as the skeleton, approximately 30% hemicellulose (heterogeneous polymer composed of different types of monosaccharides) in a bonding role [5] and approximately 25% lignin (a complex, amorphous, 3D reticulated phenolic polymer composed of phenylpropane units) in a penetrating role. Wood is gradually assembled from monomolecular cellulose (~0.52 nm), elementary fibrils (2~3 nm), microfibrils (10~30 nm), macrofibrils (~10 μm) and cell wall lamellae (S1, S2 and S3 layers in primary wall and secondary wall) by inherent physical and chemical interactions [6]. After physical modification, chemical modification or physical/chemical combination modification, it will provide an important substrate and template for the bionic preparation of high-performance, high-value and multifunctional novel materials. The further development of functional and intelligent wood is bound to create an unlimited potential for novel material fields such as selective adsorption and separation, catalyst loading, water purification, seawater desalination, photoelectric devices and sensing devices [7,8,9].

## 2. Application of Wood Composites in Water Purification

Water is the source of life and the link that binds all living beings on this planet. The rapid development of industry and the rapid growth of population have caused serious water pollution issues. More attention should be drawn to the deterioration of the global ecological environment and the scarcity of freshwater resources. Determining how to treat wastewater from different fields (metallurgy, mining, chemical industry, leather industry, batteries, etc.), nuclear energy, agriculture, shipping and so on is an important and urgent research topic [10,11,12,13,14]. Nowadays, the common materials for polluted water treatment are activated carbon, bentonite, diatomite, geopolymer, fly ash, resin and so on. However, they generally have some problems, such as high price, low treatment speed, poor recycling, poor hydrophilic pollutant removal efficiency, single pollutant type, small adsorption capacity, easily causing secondary pollution, and easy oxidation. The unique pore structure of wood is very beneficial for fluid to flow through. Meanwhile, it absorbs the tiny particles in the intercepted fluid. More importantly, it is green, light in weight, good in toughness, impact-resistant and renewable. It has great potential in the field of high-flux wastewater treatment [15,16]. Under the background of the global resource crisis, wood is increasingly being used as a raw material. After functionalization, novel wood composite filter membrane sand adsorption materials can be obtained to remove heavy metal ions, microorganisms, aromatic dyes, oil stains and other pollutants from wastewater. Apparently, this is of great and positive significance to ecological protection and resource recovery and reuse.

### 2.1. Adsorption of Heavy Metal Ions

Because of their good solubility and stability, heavy metal ions in water exhibit the characteristics of high toxicity, non-degradation and biological enrichment in the ecosystem. If water containing heavy metal ions is discharged into the environment without treatment, it will cause serious harm to human health and the safety of other organisms [17]. Nowadays, the common methods to remove heavy metal ions from industrial wastewater include chemical precipitation, lime condensation, ion exchange, reverse osmosis and solvent extraction. However, they generally have problems such as complicated operations and high costs [18]. Therefore, an ideal choice is to treat heavy metal ions with adsorbents for the deep purification of water. Moreover, an adsorbent should meet the following standards: (1) low-cost and reusable; (2) effective and rapid; (3) selective and economically feasible [19]. The microstructure of wood contains a large number of hollow cells, which are interconnected and form interconnected channels, displaying a certain water flux. Moreover, wood is a typical multi-group ligand that can purify wastewater by adsorbing various heavy metal ions: (1) O– and COO– on the wood will react with heavy metal ions (Mn^+^); (2) the negative polar bond in –OH, –NH, –OCH_3_ and –C=O in the wood will generate electrostatic attraction with heavy metal ions; (3) –OH and –COOH in the wood will exchange ions with heavy metal ions, and H^+^ will be released into the water.

Sawdust is cheap and contains cellulose and lignin, which can absorb a variety of heavy metal ions. Therefore, it has a broad application prospect in the field of wastewater treatment. Ahmad et al. [20] ground sawdust into wood powder. Then, formaldehyde was used for the methylation reaction of wood powder to produce an adsorbent. The results show that the maximum removal rates for Cu^2+^ and Pb^2+^ are 99.39% and 94.61% when the adsorption material is in a solution with successive pH values of 7.0 and 6.6. Too high or too low a pH value will reduce the adsorption capacity of materials. This is because ion exchange and hydrogen bonding are the key to the efficiency of the removal of heavy metal ions by the adsorption material. In a water environment with a lower pH value, H^+^ competes with heavy metal cations for adsorption sites on adsorption materials. In a water environment with a higher pH value, OH^−^ will form soluble hydroxyl complexes with heavy metal cations, and the electrostatic interaction between heavy metal cations and adsorption materials will be weakened. Chen et al. [6] modified fast-growing eucalyptus bark (MEUB) with a formaldehyde and sulfuric acid solution to prepare a wood adsorbent. The result shows that MEUB has different action mechanisms on Pb^2+^, Ni^2+^ and Cr^6+^ in wastewater. The adsorption of Pb^2+^ and Ni^2+^ is physical adsorption (no variation after heating). The adsorption of Cr^6+^ needs to overcome a certain activation energy, thus being classified as chemical adsorption (promoted variation after heating).

**Figure 1:**
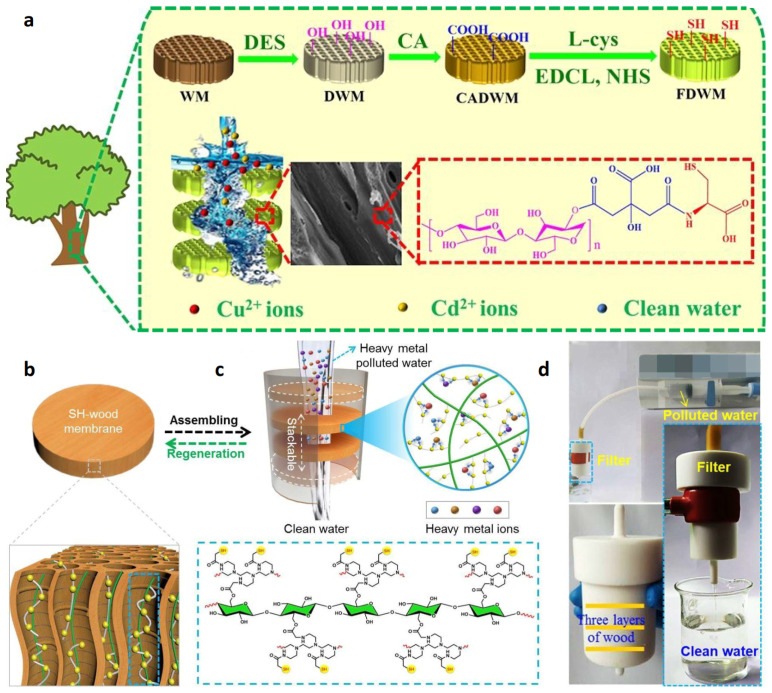
(**a**) A 3D wood microfilter for fast and efficient removal of heavy metal ions from wastewater. Schematic of SH-wood stacks for heavy metal ion removal from aqueous solution [21]. Reprinted with permission from Ref. [21]. Copyright 2020 ACS Publications Ltd. (**b**) SH-wood membrane, including a magnified drawing of its microstructure and chemical composition [22]. (**c**) The multilayer device for large-scale heavy metal ion removal. The magnified schematic shows that the heavy metal ions can combine with −SH groups when the polluted water flows through the channels of modified wood [22]. (**d**) Photos of the experimental setup for the filtration of heavy-metal-polluted water and the clean water flowing out through the three-layer SH-wood membrane [22]. Reprinted with permission from Ref. [22]. Copyright 2023 ACS Publications Ltd.

In order to improve the adsorption capacity, adsorption efficiency and selectivity of wood as adsorbent, other functional groups or inorganic nanomaterials can be further grafted or loaded in the wood channels. He et al. [21] prepared a 3D wood microfilter by modifying wood for the removal of heavy metal pollutants in wastewater. Specifically, a green deep eutectic solvent was used to remove lignin from beech wood. Then, carboxyl and sulfhydryl groups (-SH) were grafted on the surface of cellulose by sequentially using citric acid and l-cysteine. Finally, a 3D wood microfilter with an abundance of pores and adsorption sites was formed (Figure 1a). The adsorption kinetics and adsorption isotherms of heavy metal ions (Cu^2+^ and Cd^2+^) on the 3D wood microfilter were systematically investigated. The results showed that the 3D wood microfilter had a fast adsorption rate and high saturation capacity for both Cu^2+^ and Cd^2+^. Based on the advantages of easy multilayer assembly, a three-layer wood microfilter was designed to achieve the high flux rate (1.53 × 10^3^ L·m^−^^2^·h^−^^1^) and high removal efficiency (>98%) for heavy metal ions in wastewater.

Yang et al. [22] prepared a sulfhydryl functionalized wood (SH-wood) membrane with a three-dimensional mesoporous structure and low-tortuosity lumens. This SH-wood membrane serves as a multisite metal trap that achieves a high removal efficiency towards heavy metal ions from wastewater (Figure 1b,c). Benefiting from the unique microstructure of wood, the as-prepared membrane exhibits a high saturation absorption capacity of 169.5 mg·g^−1^, 384.1 mg·g^−1^, 593.9 mg·g^−1^ and 710.0 mg·g^−1^ for Cu^2+^, Pb^2+^, Cd^2+^, and Hg^2+^, respectively. Meanwhile, the SH-wood membrane can be easily regenerated at least eight times without apparent performance loss. Furthermore, an SH-wood filter with stacking multilayers was designed. Because of its high heavy metal ion absorption capability, the multilayer SH-wood filter can effectively remove diverse heavy metal ions from real wastewater, meeting the WHO standards and displaying a high flux rate of 1.3 × 10^3^ L·m^−2^·h^−1^ (Figure 1d). A further research result shows that the SH-wood membrane can be reused at least eight times. The cost of wastewater treatment is approximately USD 1 per ton, and thus, this treatment option has better economic and practical potential than traditional heavy metal ion adsorbents (activated carbon, clay, etc., displaying drawbacks such as easily being influenced by the environment, unstable treatment effect and fewer recycling times). Apparently, the SH-wood device has more development potential [23].

Cai et al. [24] synthesized UiO-66-NH_2_ (a metal–organic framework (MOF) material with photocatalytic activity) in situ in the wood channels to produce a UiO-66-NH_2_/wood composite membrane. Then, three layers of composite membranes were assembled together as a composite filter. Compared with the results of Yang et al. [22], the treatment rate of the device towards simulated wastewater containing Cu^2+^ and Hg^2+^ is 1.3 × 10^2^ L·H^−^^1^·m^−^^2^. The Cu^2+^ and Hg^2+^ removal rates of the sample are over 90%, and the treated water still meets the drinking water standard. Wang et al. [25] used pine as a template and prepared metal oxides (NiO and NiO/Al_2_O_3_) in it using the impregnation and calcination method. The result shows that NiO and NiO/Al_2_O_3_ have good adsorption towards Pb^2+^ in simulated wastewater, and their removal rates can be over 99%. Vitas et al. [26] modified beech wood with 3 mmol∙g^−^^1^ –COOH groups by optimizing the reaction conditions of esterification using anhydride. The result shows that the modified beech wood can be used as a biological adsorbent to remove 95% of Cu^2+^ from low-concentration solutions (100~500 mg∙L^−^^1^). Raman spectroscopy and energy spectrum images confirmed –COOH was mostly located on the wood cell walls. Liu et al. [27] compared a wood membrane with a pore size of several tens of microns (μm) with a polymer membrane with a pore size of 0.45 μm. Compared with the polymer membrane with a cobweb structure, the removal efficiency of the wood membrane towards Fe^3+^, Cu^2+^ and Mn^2+^ by gravity-driven filtration was improved. This is because the sponge-like structure of the polluted layer of the wood membrane prolonged the retention time of heavy metal ions. The carboxylic group (–COOH) content of the fouling layer for the wood membrane was greater than that for the polymer membrane. Additionally, the heavy metal captured more microbes on the surface of the wood membrane compared with that of the polymer membrane. Wood membranes provide a promising route to producing facile, biodegradable and sustainable membranes as a green alternative to polymer membranes in heavy metal removal from drinking water. After the pyrolysis of a urea-impregnated wood sponge in an argon atmosphere, Gu et al. [28] successfully prepared a novel adsorbent, namely a lignosulfonate (LS) functionalized g-C_3_N_4_/carbonized wood sponge (denoted as LS-C_3_N_4_/CWS). As expected, the as-prepared LS-C_3_N_4_/CWS shows excellent decontamination capability toward Pb^2+^, Cd^2+^ and Cu^2+^ with high adsorption capacities of 659.6 mg·g^−1^, 329.1 mg·g^−1^ and 173.5 mg·g^−1^, respectively, which is superior to that of most of the reported wood-based adsorbents and nanomaterials. Moreover, the LS-C_3_N_4_/CWS can be readily recovered, and it maintains a high removal efficiency after ten cycles of adsorption–regeneration, displaying excellent recyclability. Significantly, the LS-C_3_N_4_/CWS can be directly utilized as an ultrafiltration membrane to continuously treat a large volume of simulated wastewater (9550 mL·g^−1^ for Pb^2+^, 1500 mL·g^−1^ for Cd^2+^, and 8700 mL·g^−1^ for Cu^2+^). After filtration, it can result in a lower concentration than the permitted concentration in drinking water.

Compared with traditional methods, modified wood composites show better results in terms of lifetime and throughput. Thanks to the unique microstructure of wood, its efficiency of removal of heavy metal ions in wastewater can reach at least 90%. The mechanical properties of wood composites are particularly prominent as well, with excellent repeatability, strong anti-fouling ability and stable performance. Moreover, a high flux of modified wood composites, 1300~5000 L·m^−^^2^·h^−^^1^, can be maintained. Most importantly, modified wood composites are green, environmentally friendly and biodegradable.

### 2.2. Disinfection and Sterilization

According to the statistics of the World Health Organization (WHO), approximately 1.6 million people die of diarrhea due to the lack of safe drinking water and basic sanitation facilities every year. Sterilization and disinfection of drinking water can effectively prevent diseases from being spread in water. The pore structure of wood has a natural barrier to larger colonies. After combination with antibacterial nanoparticles (NPs) (e.g., Ag NPs), a wood water filter with outstanding bacteria removal ability can be made. This is because Ag enters bacterial cells in the form of particles through endocytosis and is continuously released in the form of Ag^+^. Specifically, Ag^+^ will cross-link or catalyze DNA molecules to form free radicals. Then, the proteins are denatured, the electron donors on DNA molecules are inhibited, and the DNA molecular chains break. In addition, Ag^+^ can combine with sulfhydryl and amino groups in cells, which will destroy the activity of cell synthetases. All the above procedures make bacteria and other microorganisms lose their ability to reproduce. When the bacteria die, Ag^+^ will be released and repeatedly perform the function of sterilization [29,30].

**Figure 2:**
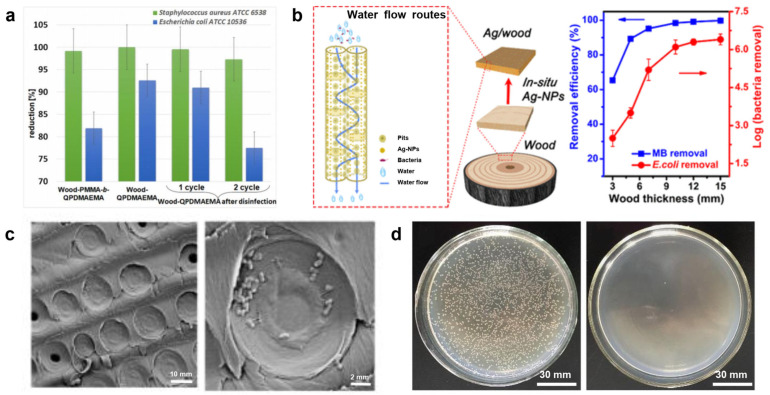
(**a**) Percent of reduction (PR) in *S. aureus* and *E. coli* culture density after incubation on modified wood blocks in reference to control wood sample [31]. Reprinted with permission from Ref. [31]. Copyright 2022 MDPI Ltd. (**b**) Filtration of model bacteria by the xylem filter [32]. Reprinted with permission from Ref. [32]. Copyright 2022 MDPI Ltd. (**c**) SEM images showing bacteria accumulated on the margo pit membranes after filtration. Scale bars are 10 mm and 2 mm [2]. Reprinted with permission from Ref. [2]. Copyright 2014 Public Library of Science. (**d**) Natural-wood- and Ag/wood-filtrated *E. coli* suspension [33]. Reprinted with permission from Ref. [33]. Copyright 2019 ACS Publications Ltd.

Macior et al. [31] used poly(methyl methacrylate) (PMMA) and poly (2-(dimethylamino) ethyl methacrylate) (PDMAEMA) to functionalize and endow wood with antibacterial properties. The antibacterial result showed that no bacterial growth was observed where the wood block was in direct contact with *S. aureus* and *E. coli* inocula. Therefore, it showed that the modified wood block had good antibacterial performance (Figure 2a). Dai et al. [32] used nano-silver, which is characterized by antimicrobial properties, to modify wood (Figure 2b). The results show that when the retention rate of silver reaches 0.324 g·m^−2^, the bacteriostatic rates of wood for Aspergillus Niger, Penicillium Citrinum and Trichoderma Viride were significantly improved, and the EX-Fr values reached 80%, 75% and 80%, respectively. Boutilier et al. [2] removed bark from pine branches, selected the part (xylem) rich in transport tissue, and inserted it into a catheter. Then, a wastewater filter was prepared based on the physical barrier of the wood structure. The result shows that the sample can filter out the bacteria in polluted water, and its removal rate exceeds 99.9%. The filtration and sterilization mainly occur in the first 2~3 mm part of the wood xylem (Figure 2c). Approximately 4 L of purified water can be obtained through 1 cm^2^ of filtration area every day, which is enough to meet a person’s normal drinking water demand. Che et al. [33] prepared an antibacterial wood filter by in situ synthesis of Ag NPs in mesoporous wood. When the mass fraction of Ag NPs in the filter is 1.25%, it can not only remove *E. coli* (6.0 orders of magnitude) and *S. aureus* (5.2 orders of magnitude) in polluted water (Figure 2d), but also remove cationic water-soluble aromatic dyes such as methylene blue (MB, 98.5%).

Electroporation sterilization technology applies a pulsed strong electric field to microorganisms and bacteria. It will destroy the cell membrane of bacteria and cause an osmotic imbalance inside and outside the cell membrane, eventually leading to the death of bacteria. There is no toxic by-product in the sterilization process. However, the high energy consumption and high risk of this technology limit its use in wastewater treatment [34]. Notably, researchers found that introducing 1D nanomaterials into conductive materials can solve the problems of energy consumption and safety [35]. Yang et al. [36] uniformly loaded Ag NPs into wood pores using the impregnation method, and the wood was further carbonized in a high-temperature tubular furnace. Then, Ag NP/carbonized wood membrane (3D Ag NP/WCM) composites with a three-dimensional mesoporous structure were obtained. The results show that the structure of nanofibers in carbonized wood is clearer. When a voltage is applied, the nanofibers will produce a peaking effect and greatly enhance the surrounding electric field, which can destroy the cell membrane of bacteria and lead to their inactivation. After electroporation, the damaged bacterial cells are more conducive to the invasion of Ag NPs in the carbonized wood and promote the sterilization process. The 3D Ag NP/WCM composite can be used in the condition of low voltage (4 V), low energy consumption (2 J·L^−^^1^) and high flux (3.8 × 10^3^ L·h^−^^1^·m^−^^2^). It has a high bacteria removal rate (over 99.999%) and good stability (over 12 h). Compared with traditional electroporation sterilization technology, the wood composite not only avoids high energy consumption, but also reduces the safety risk of operation. It is a green, economical, fast, renewable and high-flux sterilization material for water treatment. Du et al. [37] used the plate counting method to investigate the antibacterial effect of Ag@Wood. The result shows that the original wood has no antibacterial activity. In contrast, Ag@Wood exhibited the antibacterial efficiencies of 99.97%, 99.98% and 99.98% towards *E. coli*, *S. aureus* and *B. subtilis*, respectively, displaying a remarkable antibacterial activity. This is because Ag NPs in wood can bind closely to hydrosulfonyl in zymoprotein from bacteria, which could coagulate the protein, destroy the activity of bacterial synthase, and finally limit the proliferation and development of the bacteria. In addition, the dissolved Ag^+^ in Ag@Wood will further combine with bacterial membrane proteins, interfere with the synthesis of peptidoglycan and hinder the synthesis of the cell wall, leading to the leakage of substances in the bacterial membrane. Last but not least, Ag^+^ is released from the inactivated bacteria and continues its bactericidal activities.

Nowadays, traditional methods for sterilization are chlorination disinfection, ozone disinfection, heavy metal ion disinfection, etc. They work by decomposing the organic matter, bacteria and microorganisms in water through a hydroxide reaction or peroxidation. However, they usually have some issues such as cancer-causing by-products, high cost and difficult maintenance. Compared with these traditional sterilization and disinfection methods, wood composites have the characteristics of high efficiency, simplicity, stability, low cost, environmental protection and so on, and they are basically not affected by the surrounding temperature and pH. In addition, wood is green and rich in cellulose, hemicellulose and lignin, and it does not produce secondary pollution or toxic carcinogens. Moreover, the wood microstructure can be resistant to many species (e.g., *Aspergillus Niger*, *E. coli*, *S. aureus* and *B. subtilis*) and achieve a water flux of up to 3.8 × 10^3^ L·h^−^^1^·m^−^^2^. Wood composites are capable of removing at least 75% or even up to 100% of bacteria from wastewater. Therefore, wood composites have considerable development prospects in the field of sterilization and disinfection.

### 2.3. Removal of Aromatic Dyes

Printing and dyeing wastewater contains a lot of aromatic dyes, which are very difficult to remove. In addition, it also has the characteristics of dark color, high chemical oxygen demand (COD), high biological oxygen demand (BOD), complex and changeable composition, large discharge, wide distribution and difficult degradation. If the industrial wastewater is discharged without treatment, it will inevitably bring serious harm to the ecological environment due to its toxicity [38]. Therefore, removing these dye pollutants from water resources and wastewater is vital and important [39]. The natural pore structure of wood has a strong physical adsorption effect on aromatic dyes in wastewater. In addition, when the printing and dyeing wastewater flows through the pores of wood, its hydrodynamic effect is enhanced. In order to increase the time and opportunity of aromatic dye contact with active sites, functional nanomaterials or groups are loaded or grafted in the pore channels of wood.

**Figure 3:**
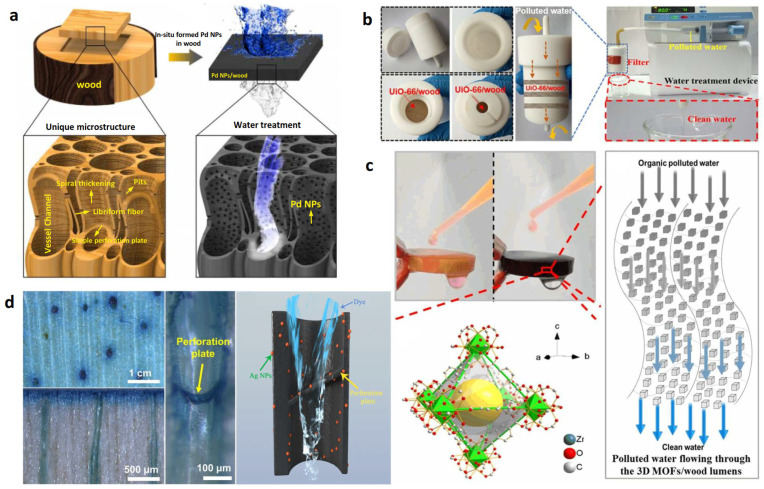
(**a**) The magnified image shows the Pd NPs in the wood channels and the color change (blue to colorless) when the MB solution flows through the Pd NP/wood membrane [16]. Reprinted with permission from Ref. [16]. Copyright 2017 ACS Publications Ltd. (**b**) Propranolol removal performance of the all-in-one three-layer filter based on the UiO-66/wood 258 membrane [41]. (**c**) Rh6G removal performance of the UiO-66/wood membrane [41]. Reprinted with permission from Ref. [41]. Copyright 2019 ACS Publications Ltd. (**d**) Water transport pathways within the wood filter [38]. Reprinted with permission from Ref. [38]. Copyright 2020 ACS Publications Ltd.

Chen et al. [16] synthesized Pd NPs in situ in basswood microchannels by using a hydrothermal method to prepare a Pd NP/wood membrane (Figure 3a). Specifically, cellulose, with rich hydroxyl groups, can immobilize Pd NPs; thus, the wood changed from yellow to black at first. This is because the plasma effect produced by Pd NPs fixed on the surface of a wood microchannel absorbs a lot of light. When wastewater containing MB flowed through the wood microchannels, MB was degraded by Pd NPs. The color changed from blue to colorless, and the MB degradation efficiency was over 99.8%. The interaction between MOFs and aromatic dyes can be used to treat different aromatic dyes in wastewater [40]. Guo et al. [41] used ZrCl_4_, terephthalic acid and acetic acid as precursors for the in situ synthesis of UiO-66 MOF nanoparticles in three-dimensional mesoporous wood using the hydrothermal reaction method to obtain a UiO-66/wood membrane (Figure 3b–d). Wood membrane filters for wastewater treatment can be obtained by changing the size and layers of this UiO-66/wood membrane according to actual needs. The results show that the flux of the filter assembled with three pieces of wood membrane is 1.0 × 10^3^ L·m^−^^2^·h^−^^1^. The removal rates for cationic water-soluble aromatic dyes such as rhodamine 6G (Rh6G), propranolol and bisphenol A exceeded 96%, offering a rapid, multi-effect and recyclable method for removing aromatic dyes in this field. Wood has an abundance of nutrient transportation channels. Meanwhile, it is also a natural water purifier. Nevertheless, the main porous structure of initial wood is not enough to effectively separate small molecules such as aromatic dye pollutants. Meanwhile, fouling in a filter will block the channels and cause poor water flux, which will restrict its large-scale application. Liu et al. [42] combined Fenton-like catalysis based on Mn_3_O_4_ loading with the microchannels of fir wood for water transfer and wastewater purification (the interfacial area was estimated to be up to 6 × 10^4^ m^2^/m^3^). The results showed that Mn_3_O_4_/TiO_2_/wood exhibited remarkable catalytic efficiency in the degradation of MB. The pollution problem in the Fenton-like catalysis process can be significantly alleviated. Goodman et al. [43] fixed graphene nanosheets (GnPs), which were treated with lignin, in basswood by vacuum impregnation to produce a GnP wood filter. When the water flux is 364 L·m^−^^1^·h^−^^1^, its MB adsorption capacity in a 10 mg·L^−^^1^ MB solution reaches up to 46 mg·g^−^^1^. Further exploration found that the aromatic dyes in the GnP wood filter and generated waste after adsorption can be effectively removed by the solvent exchange method. After five cycles of adsorption, its regeneration efficiency is still more than 80%.

However, the efficiency of the removal of high-concentration aromatic dyes by conduits in wastewater is generally low, which is the same for wood-based devices with tracheid channels [44]. In solvothermal conditions, Cui et al. [45] introduced polyoxometalate-based metal–organic frameworks (POMOFs) into natural wood (POMOF/wood) for effectively removing aromatic dyes and capturing iodine. Keggin-type POM anions with a highly negative charge were encapsulated to adjust the charge of the UiO-66 MOF, and the charge overcompensation in the POMOFs allowed them to efficiently adsorb cationic dyes. Benefiting from wood’s unique microstructure, the removal efficiencies of POMOF/wood towards MB and gentian violet (GV) (with a permeance of 1.0 × 10^4^ L·m^−2^·h^−1^·bar^−1^) reach up to 94.07% and 95.23%, respectively. Furthermore, POMOF/wood has a high capacity for capturing iodine, with a maximum adsorption of 1232.8 mg·g^−1^ in vapor. Cheng et al. [38] synthesized Ag NPs in balsa wood to prepare a dual-function Ag/wood filter that can simultaneously remove aromatic dyes and separate oil and water (Figure 3d). The results show that Ag NPs anchored on wood channels act as the catalytic sites for MB degradation in wastewater. The superhydrophilicity and underwater superoleophobicity of Ag/wood can effectively separate oil from water when the flux is 2600 L·h^−^^1^·m^−^^2^ driven by gravity. The MB removal efficiency of the Ag/wood filter (thickness: 6 mm) can reach 94.0%, and its oil–water separation efficiency is over 99%.

Liu et al. [46] used natural wood and MoS_2_ as an effective photocatalyst to remove organic pollutants from water under solar illumination. The results show that the photocatalytic degradation activity of wood/MoS_2_ composites is obviously better than that of natural wood under one solar irradiation. The improvement of photocatalytic degradation performance is attributed to (1) the increased specific surface area and (2) the catalytically active sites introduced from the edge. These factors accelerate the adsorption and degradation of organic pollutants. The synergistic effect of adsorption and photocatalysis also ensures the high versatility of the composite catalyst in the degradation of aromatic dyes. The kinetic constants of photocatalytic degradation for rhodamine B (RhB), methyl orange (MO) and MB are 0.040, 0.035 and 0.032 min^−1^, respectively. The degradation performance can be well maintained after three cycles of testing. Considering the three-dimensional mesoporous structure of wood and the catalysis of anchored Ag/AgCl NPs, Zhang et al. [47] prepared a Janus PPy@Ag/AgCl@Wood membrane that can be applied to the purification of dye-contaminated wastewater. First, the degradation capacity of Janus mesoporous wood-based membrane was evaluated utilizing MB as the model dye. A blue MB/NaBH_4_ solution (10 mg·L^−1^/100 mg·L^−1^, pH = 10) faded to colorless after permeating the membrane, showing a degradation efficiency of 86.4%. The main reason is that MB is a classic cationic dye, and cellulose in wood cell walls with hydroxyl groups usually carries many negative charges. Due to physical adsorption (e.g., Van der Waals’ force, hydrogen-bond interaction, electrostatic attraction), some MB is directly removed. The MB/NaBH_4_ solution and its filtrate showed a blue shift. It was proved that when the solution passes through the microchannels of the Janus wood, it is catalyzed by the anchored Ag/AgCl NPs. Therefore, the N-demethylation reaction occurred.

The adsorbents used for aromatic dye removal in industrial wastewater mainly include activated carbon, silicon polymers, macroporous resin and other materials with large specific surface areas. However, they have not been widely used due to their relatively high cost and poor degradation properties. In contrast, modified wood composites are green and low-cost, and their dye removal rate can reach more than 94%. At the same time, the water flux of wood composites can reach 3.0 × 10^3^ L·m^−2^·h^−1^, greatly reducing the cost during the treatment, offering a good application prospect in the field of dye removal.

### 2.4. Oil–Water Separation

Oil pollution in wastewater mainly comes from petroleum exploitation, the chemical industry, steel factories, coking workshops, gas generating stations and other industrial departments. Its mass concentration is generally 5000~10,000 mg/L. Most of these oils float on the surface of rivers and oceans and form oil films, resulting in a lack of oxygen in water bodies. Eventually, this results in the death of a large number of aquatic organisms. When oil washes up on a beach, it will cause serious harm to the waterfowls, shrimps, crabs and other creatures on the beach [48]. Wood also has a good effect on oily wastewater separation; its pit structure is very beneficial to the demulsification of oil–water emulsions. In the filtration and separation of oil–water mixtures, the wettability of the solid material plays an important role and has become an accelerator in this field. The key points in preparing special wetting materials are (1) the biomimetic construction of a hierarchical micro–nanostructure on the surface of a substrate with low/high surface energy or (2) directly using low/high-surface-energy materials to biomimetically construct a hierarchical micro–nanostructure on the surface of a substrate.

**Figure 4:**
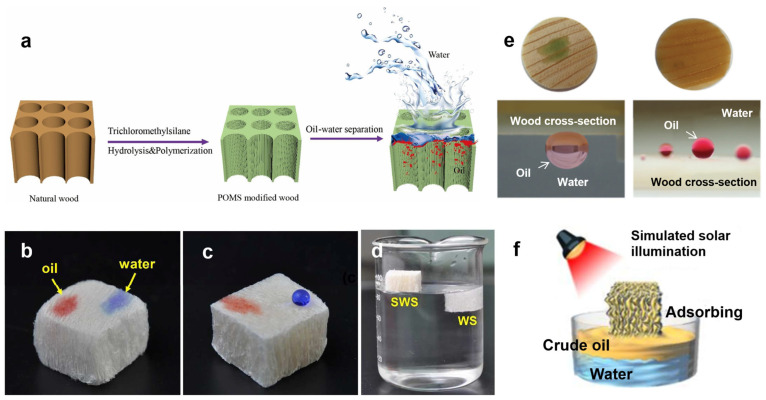
(**a**) Schematic illustration of the preparation procedure of POMS-modified wood for oil–water separation [50]. Reprinted with permission from Ref. [50]. Copyright 2020 Elsevier Ltd. Photographs of water and oil droplets on (**b**) natural wood and (**c**) silylated wood sponge (SWS) [52]. (**d**) Photographs of the SWS floating on the water surface in contrast with the wood sponge (WS) sinking in the water [52]. Reprinted with permission from Ref. [52]. Copyright 2018 ACS Publications Ltd. (**e**) Wetting properties of the wood cross-sections. Photographs of dyed light and heavy oils dripping on the wood surface underwater with high contact angles [57]. Reprinted with permission from Ref. [57]. Copyright 2017 Wiley-Blackwell Ltd. (**f**) Graphical illustration of the simulated solar illumination assisted crude oil adsorption [58]. Reprinted with permission from Ref. [58]. Copyright 2020 Elsevier Ltd.

Superhydrophobic wood nanocomposites can be obtained by removing lignin from wood, further loading nanomaterials and then carrying out polymer backfilling and silanization treatment. Then, the expected goal for oil–water separation with high efficiency, high precision and high controllability can be achieved [16,44,49]. Zhao et al. [50] modified wood with polymethylsiloxane (POMS) to obtain superhydrophobic wood materials with a water contact angle (WCA) of 153° (Figure 4a). POMS-modified wood has good oil absorption and oil–water separation performances. However, the accurate filtration efficiency and reusability of POMS-modified wood still need further discussion. Aside from cellulose, which is rich in hydroxyl groups, lignin and hemicellulose also exist in wood. They also contain -NH_2_ and -OH groups, resulting in the good hydrophilicity of wood. When wood is soaked in water, a hydrophilic and oil-repellent water film is formed on its surface. When an oil–water mixture is dripped on a wood surface, the water permeates into the wood, while the oil is excluded, showing a good superoleophobicity underwater. Wang et al. [51] prepared a Ag NP/wood membrane using a simple one-step hydrothermal method. The results show that the loading of Ag NPs increases the micro/nanoroughness of wood, which is beneficial to the demulsification of oil-in-water emulsions. Even after 10 cycles of testing, the oil-in-water emulsion separation efficiency was still over 90%. Within 5 min, its photocatalytic degradation rate for MB was 97.21%. Guan et al. [52] selectively removed the lignin and hemicellulose of wood without further filling treatment. After modification by methylsilylation (Figure 4b–d), a highly porous hydrophobic wood sponge (SWS) with enhanced mechanical elasticity, low density and an oleophilic property was directly prepared. The “wood sponge” has a high oil absorption capacity of 41 g/g and excellent recovery capacity. Moreover, the assembled filter can continuously separate oily wastewater with the flux of 84.7 L·h^−^^1^·g^−^^1^.

After selective removal of hemicellulose and lignin, the basic framework of cellulose with hierarchical high porosity and low density can be prepared easily [53,54]. Fu et al. [55] used a NaClO_2_ solution to remove the lignin of balsa wood. After freeze-drying, a porous delignified wood template with high hydrophilic and oleophobic properties was obtained. Then, it was impregnated with epoxy resin/amine/acetone solution. After curing, a hydrophobic and oleophilic wood composite with a unique pore structure was prepared. The product shows an outstanding compression strength (263 MPa) and oil absorption effect (15 g/g), and it can absorb oil pollution on and under the surface of water at the same time. Wang et al. [56] coated one side of delignified wood with a dodecyl mercaptan solution. After ultraviolet radiation induction, a Janus wood membrane with asymmetric wetting properties and unidirectional water transmission was prepared, and it was suitable for selectively separating the mixtures of light oil/water and heavy oil/water (the separation efficiency was higher than 99.3%). Blanco et al. [57] directly used spruce (thickness: 1 mm) for oil–water separation. Under gravity, its flux (3500 L·m^−2^·h^−1^) and efficiency (>99%) for separating oil–water mixtures were investigated (Figure 4e). After the loading of Ag NPs in wood, superhydrophilic and underwater superoleophobic wood nanocomposites were prepared, the surface hydrophilicity of which could be further improved. After in situ auxiliary modification of photothermal materials (graphene) and transparent hydrophobic materials on delignified and hemicellulose wood, Chao et al. [58] prepared a compressible and resilient photothermal wood aerogel (Figure 4f). Based on the characteristics of the decrease in viscosity and increase in fluidity of crude oil with an increase in temperature, as well as the capillary force of the wood aerogel, its adsorption capacity for crude oil can reach 0.801 g·cm^−3^. The transparent hydrophobic coating of the aerogel endows it with selective adsorption of the oil phase. An intelligent infiltration effect of the crude oil phase at different temperatures can be realized at the same time. Moreover, the adsorbed crude oil can be released and collected by simple mechanical extrusion. The material can be compressed and recycled more than 10 times. It solves the problems of the high energy consumption, complicated separation process, unsatisfactory treatment effect and secondary pollution caused by traditional oil–water separation methods (gravity separation, centrifugation, air flotation, in situ combustion, bioremediation and flocculation, etc.).

Zhu et al. [59] fabricated a superhydrophobic methyltrimethoxysilane (MTMS)-modified wood aerogel (M-WA) using the vapor deposition method for oil–water separation. In order to build micro–nanoroughness and sufficient pores, balsa wood was chemically treated to remove lignin and hemicellulose and modified by MTMS vapor deposition. The superhydrophobic M-WA shows a high WCA of 151.8°. M-WA can selectively adsorb oil from an oil/water mixture, and its adsorption capacity for dichloromethane reaches 25.1 times its own weight. In addition, M-WA can be used as a filter to continuously separate an oil–water mixture, and its separation efficiency reaches 99.1%. Even after 20 cycles, the separation efficiency still remained at 98.5%, showing excellent recyclability. Chen et al. [60] proposed a superhydrophobic wood with excellent Joule heat and demulsification. Specifically, a carboxyl-modified carbon nanotube (cCNT) coating provides the conductive pathways, polyethylenimide (PEI) is used to support the demulsification procedure and polydimethylsiloxane (PDMS) is subsequently used for hydrophobic modification. A superhydrophobic wood with excellent Joule heat and demulsification performance was proposed. The as-prepared superhydrophobic PDMS/PEI-cCNT wood (WCA-155°) can withstand 50 cycles of compression tests under 40% strain. Oil can be separated and recovered from an oil–water mixture by an absorption–extrusion process. Due to the electrostatic interaction of PDMS/PEI-cCNT wood, a water-in-oil emulsion can be quickly demulsified with a separation efficiency greater than 99.7%. After the simple removal of lignin and hemicellulose from natural wood, cellulose nanofibers with hydrophilicity were prepared, and they were further used to produce a flexible cellulose aerogel with lamellar architecture. Wu et al. [61] used the superhydrophilicity of balsa-based cellulose aerogel to separate various aqueous oils. The separation efficiency of cellulose aerogel for immiscible water-based oil and water-in-oil emulsions can reach 99.97% and 98.45%, respectively. Shi et al. [62] prepared elastic wood (E-Wood) and then endowed it with excellent oil resistance by in situ polymerization of pyrrole for it to serve as an O/W emulsion filter. The mechanism by which PPy-E-Wood separates O/W emulsions is that water can easily wet and penetrate a PPy-E-Wood membrane and form a water film on its surface. In the separation, water quickly passes through PPy-E-Wood. However, non-polar micron oil droplets are repelled by the water film formed on the PPy-E-Wood surface. Therefore, the oil droplets are trapped at the top of PPy-E-Wood. Then, these larger oil droplets can be easily separated from the PPy-E-Wood surface by a simple rinse treatment. Its separation efficiency towards O/W (H/W and D/W) emulsions can be over 98%. The filtration is driven by gravity without external pressure. Even after 10 cycles of separation, its O/W emulsion separation efficiency still remained at 97.8%. Specifically, when PPy-E-Wood is applied to oily wastewater and oil-spilled seawater, it can effectively avoid the blockage of its exchange channels caused by oil sticking to its surface or bottom, resulting in the loss of solar evaporation performance.

Traditional oil–water separation technologies include the adsorption method, skimming method, gravity separation method, biological treatment method, air flotation method and centrifugal separation method. Most of these methods have low separation efficiency and poor selectivity. In particular, their separation effects are unsatisfactory for oil–water emulsions with a high dispersion and low concentration of oil droplets (droplet size of dispersed phase less than 20 μm). In addition, the excessive input cost leads to serious resource consumption. Wood composites can be made by modifying wood to make them have excellent hydrophilicity or lipophilicity. The separation efficiency and water flux of some wood composites can reach 99% and 3500 L·m^−2^·h^−1^, respectively. After 20 cycles, some wood composites still show a water/oil separation efficiency of 98.5%. In addition, some wood composites can withstand 50 cycles of compression tests under 40% strain.

## 3. Application of Wood Composites in Solar Desalination

The wood composites discussed in the previous section can be applied to wastewater purification without the interference of external factors (e.g., solar energy). Thanks to their structural advantages, wood composites enable simple adsorption and filtration. However, when the salt content in brine is too high, it is difficult to desalinate seawater and obtain clean freshwater by simple adsorption or filtration of wood composites. The desalination of wood composites requires the input of external energy, for which clean and abundant solar energy is generally used. The specific process includes four steps: light absorption, photothermal conversion, interface evaporation and condensation collection. While wood itself has poor photothermal conversion performance, after modification with photothermal materials, wood composites can obtain good photothermal conversion and evaporation efficiency. Notably, water molecules are present in the liquid state in the process of wastewater purification using wood composites discussed in the previous section. This section discusses the application of wood composites in the solar interfacial evaporation of seawater with high salinity. Specifically, water molecules in seawater absorb heat from sunlight and then transform from the liquid state to the vapor state. Afterward, the desalting steam is condensed and collected as liquid freshwater. This process is the transformation of water molecules from the liquid state to the vapor state and then to the liquid state. This section focuses on the structural design of wood composites in solar desalination.

As a clean and renewable energy source, solar energy has a very broad application prospect in alleviating the shortages of energy and water resources. In solar desalination, sunlight is first captured by photothermal materials and then converted to heat. Further, the latent heat of the phase change (seawater from liquid to gas) can be overcome by the converted heat. Generally, the photothermal material is separated from the water body by a material with low thermal conductivity because the more converted heat is used to heat the fluid, the less heat is lost in this process. Meanwhile, the bottom water passes through the conduits in the substrate with low thermal conductivity and is further pumped to the top of the photothermal layer by its capillary force. Trees are the most extensive resources in the world [37,62,63], the conduits of which are beneficial to water transportation. Because of its outstanding transpiration capability, water can be pumped to a height of more than 100 m. Therefore, wood cut perpendicular to the growth direction is an ideal seawater desalination material. Moreover, the transpiration and sunlight absorption inside trees occur through xylem conduits and cavities [64]. Ninety percent of water absorbed by trees is emitted into the air through transpiration. The thermal conductivity of wood is very low and highly anisotropic along (0.35 W·m^−1^·K^−1^) and perpendicular (0.11 W·m^−1^·K^−1^) to its conduits, which is very beneficial for isolating unnecessary heat exchange between a photothermal layer and seawater, thus improving the heat management and evaporation performance.

The traditional methods for seawater desalination include multi-stage flash evaporation (MSF) and multi-effect distillation (MED), as well as reverse osmosis (RO) technology. However, most of them have serious shortcomings including a high dependence on fossil resources; energy consumption; greenhouse gas emissions; high technical requirements; high costs for investment, equipment operation and maintenance; less actual return; and even secondary pollution caused by the large consumption of chemicals and detergents. Wood composites with good photothermal conversion properties with different structural designs such as directly carbonized wood, wood/carbon nanomaterial composites, wood/semiconductor composites, wood/polymer composites and wood/precious metal composites have been prepared. In addition, sunlight provides an almost infinite source of clean heat energy. With different structural designs, a high evaporation rate (1.351~4.31 kg·m^−2^·h^−1^) and photothermal conversion efficiency (87.4~122.2%) of wood composites have been achieved in solar desalination. Experimental data show that the photothermal layers on these wood composites after acid/alkaline and seawater immersion for 100 h and ultrasonic treatment for 2 h have no obvious changes. Apparently, these wood composites are extremely durable. Even after 100 cycles of freeze–thaw testing, the water evaporation rate of wood composites did not decrease significantly.

### 3.1. Directly Carbonized Wood

Wood emerges as a sustainable and promising precursor for carbon materials owing to its natural abundance and superb properties. The preparation of carbon materials with different morphologies (dots, spheres, nanowires, films and other 3D nanomaterials) and porous structures (disordered and ordered) is of great significance for various fields (e.g., adsorption, capacitors, catalysts, solar cells, sensors) [65]. Xue et al. [66] cut wood into cylinders and put them on an alcohol flame for carbonization. Then, they were immersed in cold water for rapid quenching. Finally, wooden cylinders with carbonized surfaces were obtained. Under 1 kW·m^−2^, their thermal conversion efficiency was as high as 72%. Also inspired by tree transpiration, Zhu et al. [19] designed a solar evaporation device with a double-layer structure. Firstly, wood was cut perpendicular to the wood growth direction and simply carbonized at 500 °C for 0.5 min. The thickness of the carbonized layer was only approximately 3 mm on the surface of the wood, which could be directly used for solar-driven seawater desalination. The results show the following: (1) The upper surface of carbonized wood can absorb 99% of light. (2) Under 10 kW·m^−2^, its photothermal conversion efficiency is 87%. (3) Under 10 kW·m^−2^, the amount of water evaporation increases linearly. (4) After 100 h of irradiation under 5 kW·m^−2^, the sample can still be used stably. (5) The sample can remain stable in seawater for a long time, and there is no salt accumulation on its surface. (6) Water can be directly extracted from the ground (sand and soil).

**Figure 5:**
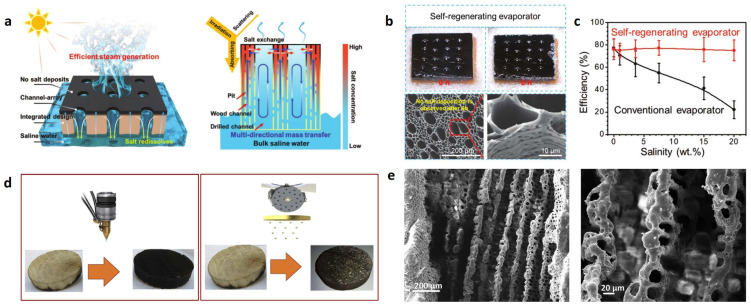
(**a**) Schematics of solar desalination by interfacial evaporation [67]. (**b**) Salt-free surface of the self-regenerating evaporator after 6 h of continuous testing in 20 wt% NaCl solution under one solar irradiation. SEM images showing the salt-free surface of self-regenerating evaporator [67]. (**c**) The influence of salt concentration on the steam generation efficiency of conventional and self-regenerating evaporators [67]. Reprinted with permission from Ref. [67]. Copyright 2019 Wiley-Blackwell Ltd. (**d**) Laser-treated wood (LT-Wood) and gold deposition wood (Au-Wood) [69]. (**e**) Laser treatment creates grooved patterns on the wood [69]. Reprinted with permission from Ref. [69]. Copyright 2020 Elsevier Ltd.

Kuang et al. [67] also used the above method to carbonize wood. The difference is that they used an electric drill to drill holes in the wood before carbonization (Figure 5a–c). Then, the carbonized layer on the wood surface was polished with sandpaper and then placed in 20 wt% NaCl solution for 6 h under one solar irradiation. The surface of the sample without drilled holes was completely covered with precipitated salt. However, there was no obvious salt deposition on the surface of the sample with drilled holes, displaying an excellent self-desalting ability. This is due to the rapid salt exchange among the pits on wood cell walls, wood microchannels and millimeter-sized drilling channels on wood. The increased salt concentration in the natural wood channel can be diluted at any time. Therefore, it will not block the steam discharge channel during the evaporating procedure of concentrated brine. It is always stable in long-term evaporation and has excellent performance under 1~5 solar irradiations. Its evaporation rate was 6.4 kg·m^−2^·h^−1^ when it was placed in high-salinity water (15 wt%) under six solar irradiations, showing excellent stability and durability. Chen et al. [68] developed a multifunctional solar evaporator composed of carbonized wood and biomass hydrogel modified by MXene. The evaporation rate and efficiency of the product were 3.71 kg·m^−2^·h^−1^ and 129.64%, respectively. Under two kinds of solar irradiation, the open circuit voltage is 1.8 mV. The high performance of the evaporator stems from (1) the high water transmission of natural wood structure, (2) the high solar absorption and heat conversion efficiency enhanced by carbonized wood and MXene, (3) the regulation of evaporation enthalpy and surface energy by MXene and (4) the temperature adjustment by the low-thermal-conductivity structural system.

Ghafurian et al. [69] utilized the natural water transport tissue of high-porosity wood (poplar) to maximize solar utilization for water vaporization. The inherent high porosity, low thermal conductivity and rapid capillary action of wood make it attractive in solar desalination. Natural wood cannot absorb broadband sunlight well. Therefore, these authors compared several innovative and potentially scalable technologies for wood surface modification, including laser carbonization, gold nanolayer deposition and their combination (Figure 5d). The use of a high-power laser is a fast and accurate method for engraving, cutting and carbonizing wood, enabling groove patterns to be generated rapidly and controllably without destroying the wood samples (Figure 5e). Under 3 kW/m^2^, the best performance of gold coating carbonized by a hot plate is 4.02 kg/m^2^·h, which is twice that of untreated sample (~2 kg/m^2^·h), and the best performance of gold nanocoating is 3.54 kg/m^2^·h. In addition, the performances of samples after 10 cycles of thermal treatment were investigated. The result showed that these samples could evaporate stably at the speed of 3.3 kg/m^2^·h. It can be concluded that surface modification provides wood with a scalable high performance. Therefore, fast-growing wood can be widely used in solar seawater desalination and/or low-temperature steam generation.

### 3.2. Wood/Carbon Nanomaterial Composites

Nanomaterials usually exhibit some unique electronic and optical properties. For instance, in the allotrope of a carbon nanotube, almost every band in the solar spectrum can excite electrons due to its large number of conjugated π bonds. This leads to various π-π* transitions inside and a dark appearance. Meanwhile, when the incident light energy matches the electron transition in the molecule, the electron will absorb the light and rise from the highest occupied molecular orbital (HOMO) to the lowest unoccupied molecular orbital (LUMO). After absorbing these energies, electrons relax through electron–phonon coupling. The carbon allotrope not only absorbs the light, but also transfers the energy of excited electrons to the atomic lattice in the vibration mode. Macroscopically, it will increase the temperature of the material [70]. In addition, other carbon photothermal nanomaterials (such as carbon nanotubes (CNTs) [71], carbon dots (CDs) [72], graphene [73], graphene oxide (GO) [74] and reduced graphene oxide (rGO) [75]) are also promising photothermal materials for interfacial water evaporation.

**Figure 6:**
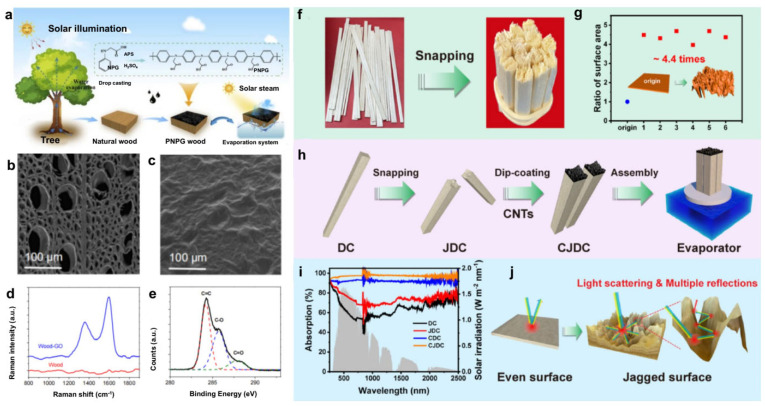
(**a**) Fabrication of PNPG wood solar evaporator [54]. SEM images of wood cross-section without (**b**) and with (**c**) GO on the surface of microporous structure [54]. Reprinted with permission from Ref. [54]. Copyright 2020 ACS Publications Ltd. (**d**) Raman spectrum of wood with (blue) and without (red) GO flake coating on wood surface [74]. (**e**) XPS spectra of wood and wood-GO composite [74]. Reprinted with permission from Ref. [74]. Copyright 2017 ACS Publications Ltd. Digital images of (**f**) pristine DC and JDC. (**g**) Top surface area ratios of JDC to DC. (**h**) Fabrication of CJDC evaporator. (**i**) Solar absorption spectra of DC, JDC, CDC and CJDC. (**j**) Schematic illustration of jagged surface for enhancing solar absorption [78]. Reprinted with permission from Ref. [78]. Copyright 2023 Elsevier Ltd.

Taking advantage of the low curvature of the pore structure and the anisotropic thermal conductivity of wood, Chao et al. [54] used delignified wood as a substrate and carbon dots with a photothermal effect prepared using the removed lignin to realize a solar evaporation system with an all-wood composition (Figure 6a–c). The results show that the evaporation rate and photothermal conversion efficiency of the solar evaporation system are 1.09 kg·m^−2^·h^−1^ and 79.5% under 1 kW·m^−2^. Liu et al. [74] obtained a wood-GO double-layer composite by dropping graphene oxide on the cross-section of wood. Under 5 kW·m^−2^, the wood-GO composite and original wood were irradiated in dry and wet states. The results showed that wood-GO had a great temperature increase (Δ_dry_ = 43 °C, Δ_wet_ = 33 °C), while the original wood changed little (Figure 6d,e). A wood-GO composite was placed in simulated seawater with a salinity of 3 wt%. Under 12 kW·m^−2^, its temperature reaches 67 °C in the first few seconds and remains constant. The generation of water vapor can be clearly seen on its surface. Compared with the original wood (10.08 kg·m^−2^·h^−1^ and 59.5%), its evaporation efficiency and photothermal conversion efficiency can reach 14.02 kg·m^−2^·h^−1^ and 82.8%, respectively. It not only realizes the effective utilization of renewable solar energy, but also realizes the full utilization of the internal circulation of wood-based materials.

To make use of the directional arrangement structure of natural wood fibers, Chao et al. [76] selectively removed lignin and hemicellulose and further modified the retained cellulose with photothermal coating (rGO) to prepare a wood-derived aerogel. Under 1 kW·m^−2^, the material was hung between seawater tanks to carry out a “connecting bridge” seawater desalination. The results show that the evaporation rate and photothermal conversion efficiency are 1.351 kg·m^−2^·h^−1^ and 90.89%, respectively. Compared with the traditional “close contact” solar evaporation, it avoids the heat loss and the decrease in light energy utilization rate caused by the close contact between photothermal materials and the water phase. It greatly improves its light energy utilization rate. Through the incomplete combustion of a paraffin candle flame, Hu et al. [77] constructed carbon nanoparticles on the surface of wood to prepare a novel double-layer solar evaporator. The results show that the carbon nanoparticle/wood composite with an interconnected porous structure has broadband and high light absorption (93~97%) and can convert solar energy into heat energy to heat the interface between water and air. Its principle is that water is effectively transported from the wood bottom to the surface of carbon nanoparticles by capillary force for sufficient evaporation water supply. Meanwhile, the abundant hydrophilic groups of wood form hydrogen bonds with water molecules, which weakens the hydrogen bonds between intermediate water molecules. Under 1 kW·m^−2^, the water evaporation enthalpy is reduced by approximately 26%, while the evaporation rate and conversion efficiency are 2.06 kg·m^−2^·h^−1^ and 90%, respectively, displaying good long-term stability, strong self-cleaning ability, and strong acid and alkali resistance.

Zhang et al. [78] used CNTs to decorate disposable hydrophilic wood chopsticks, which were assembled into a 3D array for efficient water evaporation and purification. Figure 6f shows the fabrication and solar absorption performance of CNT-functionalized disposable wood chopstick (CDC) and CNT-decorated jagged disposable wooden chopstick (CJDC) evaporators. Firstly, the chopsticks were broken into fragments with a 3D serrated top surface. Compared with a traditional flat surface, its surface area is significantly increased (Figure 6h). In order to provide jagged disposable wood chopsticks (JDCs) with photothermal conversion capability, solar thermal CNTs were coated on the surface of JDCs using the impregnation method. Then, JDCs with high photothermal performance were obtained. To increase the top and side surface areas, a single CJDC unit was assembled into an integrated 3D evaporator for seawater desalination and water purification (Figure 6i). As shown in Figure 6j, a CJDC has a higher absorption of sunlight than a CDC. The CJDC can capture the incident sunlight more effectively by multiple reflections and scattering on its surface. The exposure height of the disposable chopstick evaporator decorated with CNTs was 4 cm. The evaporation rate and energy efficiency of the wood composites were 3.70 kg m^−2^·h^−1^ and 122.2%, respectively. They have wide application prospects in solar-driven seawater desalination and industrial wastewater purification.

### 3.3. Wood/Semiconductor Material Composites

**Figure 7:**
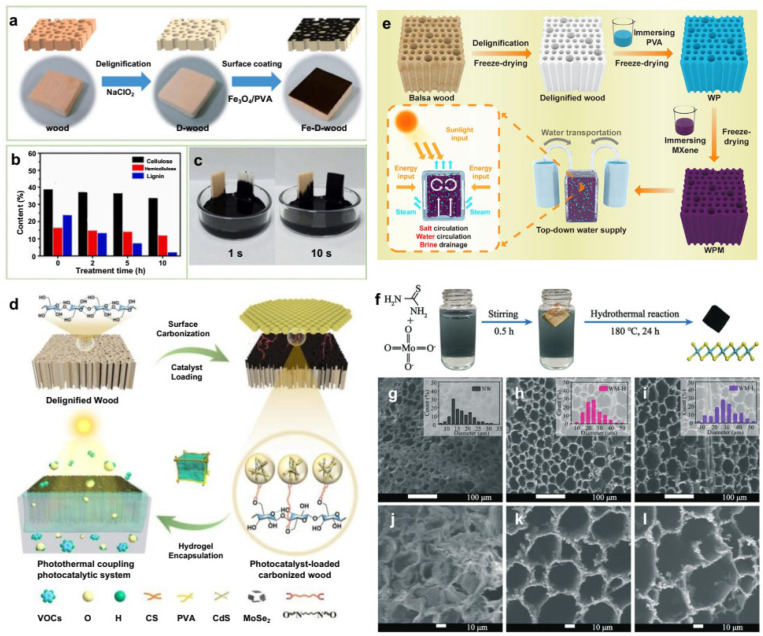
(**a**) Design and fabrication of Fe_3_O_4_/PVA-coated wood, (**b**) lignin content of wood over 10 h treatment with NaClO_2_, and (**c**) photos of pen ink transportation in natural wood (left) and delignified wood (right) [79]. Reprinted with permission from Ref. [79]. Copyright 2021 Elsevier Ltd. (**d**) Synthetic schematic illustration of the wood-based hydrogel coating with CdS-MoSe_2_ for solar steam generation and hydrogen energy conversion [82]. Reprinted with permission from Ref. [82]. Copyright 2022 ACS Publications Ltd. (**e**) Preparation of WPM evaporator and its assembly with a top-down water supply device for solar steam generation and desalination [83]. Reprinted with permission from Ref. [83]. Copyright 2023 Elsevier Ltd. (**f**) Fabrication of MoS_2_-coated wood. (**g**–**l**) SEM image of wood samples [84]. Reprinted with permission from Ref. [84]. Copyright 2021 Environmental Science Ltd.

The band gap of a semiconductor material can determine its light absorption ability. When the light strikes the semiconductor surface, the concentration of carriers (electrons or holes) in the energy band will increase continuously. When the excited electrons jump back to the low-energy state, the energy will undergo a non-radiative relaxation process. Specifically, the photon energy will be converted into heat energy, thus affecting the photothermal conversion capability. Song et al. [79] coated Fe_3_O_4_/PVA on the surface of delignified basswood to obtain an Fe_3_O_4_/PVA/wood evaporator (Figure 7a). The wood was treated with NaClO_2_ to remove hemicellulose and lignin for at least 10 h, while the cellulose content was basically preserved intact (Figure 7b). Delignified wood has better ink transferability than original wood (Figure 7c). In addition, delignified wood has better hydrophilicity than original wood. Polyvinyl alcohol (PVA) enhances the bonding force between a wood matrix and a semiconductor. Under 1 kW·m^−2^, the surface temperature of natural wood increased from 26 °C to 34 °C within 10 min, while the equilibrium temperature of Fe_3_O_4_/PVA/wood reached 63 °C. Compared with the wood composite without delignification, Fe_3_O_4_/PVA/wood shows a higher temperature. Fan et al. [80] embedded a hydrogen evolution semiconductor material (CdS) and photothermal material (MoSe_2_) into porous delignified wood simultaneously to prepare a wood-mixed hydrogel. It can be used for water purification, hydrogen production and seawater desalination. By optimizing the structure and process design, the efficient pollutant removal, hydrogen production and steam generation of the product can be realized. Under one solar illumination, its hydrogen evolution rate, solar evaporation rate and energy conversion efficiency are 9.7 mmol g^−1^·h^−1^, 1.92 kg·m^−2^·h^−1^ and 90.7%, respectively. The hydrogel packages the photocatalytic system, which can effectively prevent the evaporation of toxic volatile organic compounds (VOCs) and retain the ability of continuous and efficient steam production.

He et al. [81] soaked various woods (such as beech, cedar, pine, ash, oak, poplar and cudgel) in a tannic acid (TA) solution to obtain wood-TA. Afterward, wood-TA was immersed in Fe_2_(SO_4_)_3_ solution to obtain wood-TA-Fe^3+^. However, a polypropylene (PP) porous membrane, polyester fabric and polyurethane (PU) sponge in the same condition are blue-gray rather than black. After comparing the SEM images of original poplar, wood-TA and wood-TA-Fe^3+^, it was found that many nanonodes appear in wood-TA-Fe^3+^ due to the coordination between the doped TA and Fe^3+^. The rough wood surface and abundant pore structure further reduce the energy loss caused by light reflection. The results show that the photothermal layer of the sample after acid/alkaline and seawater immersion for 100 h, ultrasonic treatment for 2 h and 100 cycles of freeze–thaw testing has no obvious change. For complex water quality, it can effectively prevent oil droplets from adhering to the material surface, which will avoid the blocking of its waterway and a decrease in evaporation performance. In addition, the researchers also cut many grooves on the wood surface to evaluate wood-TA-Fe^3+^ with an uneven surface. The results show that the water evaporation rate of wood-TA-Fe^3+^ with surface modification reaches 1.85 kg·m^−2^·h^−1^, which is 4 times that of wood-TA-Fe^3+^. Solar interfacial evaporation is considered as a promising strategy for solar-driven seawater desalination and industrial wastewater purification. Yan et al. [82] used delignified wood (DW) as a water transport substrate and lignosulfonate (LS)-modified nickel disulfide (NiS_2_) as a light absorber (LS-NiS_2_) to prepare an efficient LS-NiS_2_/DW evaporator (Figure 7d). The results show that LS-NiS_2_ has a high absorption rate (>95%) and photothermal conversion efficiency in a wide wavelength range. Therefore, the evaporator has a higher solar energy utilization rate. On the other hand, the hydrophilicity of DW facilitates the activation of water. The evaporation enthalpy of LS-NiS_2_/DW (1274.4 kJ·kg^−1^) is lower than that of pure water. Under one solar irradiation, the evaporation rate of LS-NiS_2_/DW reaches up to 2.80 kg·m^−2^·h^−1^, and its evaporation efficiency reaches 87.4%. It is worth noting that LS-NiS_2_/DW shows a high evaporation rate (2.42~2.69 kg·m^−2^·h^−1^) in simulated seawater. Even after 24 h, no salt crystals are formed on its surface.

In order to realize high-yield, high-salinity and long-term seawater desalination, Hu et al. [83] enhanced the hydrophilicity of balsa wood by delignification. Vacuum-assisted impregnation of polyvinyl alcohol (PVA) and a special top-down water supply device were adopted. Figure 7e illustrates the fabrication procedure for 3D PVA/MXene-decorated wood (WPM), which serves as a photothermal water evaporator with a top-down water supply design for solar-driven evaporation of seawater and high-salinity water. The presence of MXene nanosheets facilitates solar light absorption and solar–thermal conversion. Therefore, the evaporation rate of the PVA/MXene-decorated wood (WPM) solar evaporator under 1 kW·m^−2^ is as high as 4.31 kg·m^−2^·h^−1^. During the desalination of a 25 wt% NaCl solution for 8 h, its evaporation rate reached 3.83 kg·m^−2^·h^−1^. A sufficient down-top water supply dilutes the saline water with a high concentration on the surface of the WPM evaporator, thus avoiding salt deposition in the long-term solar-driven desalination process. The result shows that its average evaporation rate in 15 wt% NaCl solution under solar irradiation for more than 100 h is 4.24 kg·m^−2^·h^−1^. He et al. [84] reported a 3D wood membrane coated with MoS_2_ (WM-H, with S trap) for effective seawater desalination. The MoS_2_ was synthesized using thiourea and ammonium molybdate tetrahydrate (Figure 7f). High-resolution SEM images show the structural changes (Figure 7g–l). The pore size of NW is mainly distributed at approximately 10~25 μm. The specific surface area (SSA) increases from 0.22 m^2^·g^−1^ (NW) to 9.92 m^2^·g^−1^ (WM-H) and 10.45 m^2^·g^−1^ (WM-L). The adsorption/desorption capacity of WM-H and WM-L is much higher than that of NW. After treatment, the pore structure of the wood samples becomes regular. The pore diameters of WM-H and WM-L (Figure 7g–i) increased to approximately 10~40 μm and 10~50 μm, respectively. Therefore, the vertically arranged channels of wood can rapidly provide water for its heating surface, which could support the high rate of steam generation. This is due to rapid water diffusion and powerful capillary pumping. The excellent photothermal MoS_2_ provides enough heat for water evaporation. Under 1 kW·m^−2^, MoS_2_-coated wood with S defects has an excellent evaporation rate and heat conversion efficiency of 1.46 kg·m^−2^·h^−1^ and 82.5%, respectively. The adsorption peaks of Na^+^ on both sides of defective MoS_2_ are respectively 2.17 and 1.49 times higher than those for MoS_2_ without S defects.

### 3.4. Wood/Polymer Composites

**Figure 8:**
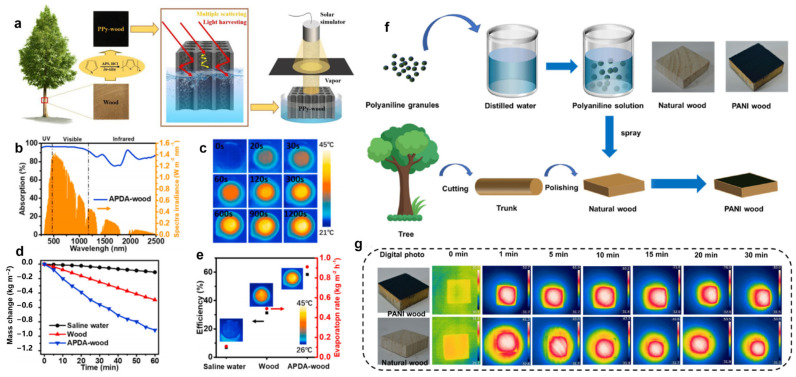
(**a**) Schematic illustration of the PPy-wood for solar steam generation [14]. Reprinted with permission from Ref. [14]. Copyright 2020 Royal Society of Chemistry Ltd. (**b**) Light absorption spectra of APDA-wood ranging from 250 nm to 2500 nm in wet state [90]. (**c**) Time-dependent IR images of the APDA-wood under 635 nm with the irradiation of 1 kW/m^2^ [90]. (**d**) Water evaporation rates of saline water, saline water with wood and APDA-wood under 1 kW/m^2^ [90]. (**e**) Solar efficiency and evaporation rate of saline water, wood and APDA-wood under visible light [90]. Two differently colored arrows indicate the scales on each side. Reprinted with permission from Ref. [90]. Copyright 2021 Elsevier Ltd. (**f**) Preparation diagram of PANI-wood [93]. (**g**) Infrared image of surface temperature of dry natural wood and PANI-wood under 1 kW/m^2^ [93]. Reprinted with permission from Ref. [93]. Copyright 2021 Elsevier Ltd.

Polydopamine (PDA) can be prepared by the self-polymerization of dopamine monomers under alkaline conditions. It has good adhesion and exhibits an absorption spectrum from ultraviolet light (UV) to near-infrared light (NIR). Therefore, it is an ideal photothermal coating material [85,86,87] that can evenly and stably adhere to the surface of a wood cavity on the basis of not blocking the pore channels. Polypyrrole (PPy) has good biocompatibility and environmental stability [14] and exhibits a high absorption rate of 90.8% in the whole solar spectrum [88,89]; it can be combined with the hydroxyl groups of cellulose through hydrogen bonds. The results show that PPy-wood shows almost full-spectrum light absorption and low incident angle sensitivity in the spectral range of 250~2500 nm (Figure 8a). Zou et al. [90] mixed arginine and a dopamine solution to obtain a black precipitate (APDA), which was coated on the surface of camphor wood to prepare APDA-wood (Figure 8b–e). Compared with traditional PDA, APDA has a narrower band gap and stronger light absorption capacity, in accordance with density functional theory (DFT). In addition, APDA has no obvious luminescence under the excitation of 365 nm, 500 nm and 808 nm, which indicates that the non-radiative transition is dominant. That is, the light absorbed by APDA will be converted into heat more quickly and effectively. Under one solar irradiation, the surface temperature of APDA-wood rises faster than that of pure wood and water. The surface temperature of APDA-wood can reach 38 °C within 5 min and stabilize at 40 °C, and the evaporation rate can reach 0.91 kg·m^−2^·h^−1^. In order to verify the actual seawater desalination, 3.5 wt% NaCl solution was used to simulate seawater. The results show that Na^+^ in simulated seawater decreased by approximately 4 orders of magnitude after APDA-wood desalination. The concentrations of Ca^2+^, Mg^2+^ and K^+^ also decreased by at least 2~3 orders of magnitude, meeting the standards of the US Environmental Protection Agency (EPA) and the WHO. Even after 100 cycles, the water evaporation rate of APDA-wood did not decrease obviously.

In addition, the physical characteristics of wood endow PPy-wood with excellent heat insulation and water transmission performance. After the absorption of a pyrrole solution and a mixed solution of APS (ammonium sulfate) and HCl, Huang et al. [91] prepared black PPy-wood. In the comparison of the optical characteristics of PPy-wood and original wood in the spectral range of 250~2500 nm, the light absorption rate of the original wood (44.9%) was much lower than that of PPy (90.8%). More importantly, under the synergistic effect of PPy and wood, the light absorption rate of PPy-wood in the whole spectral range is as high as 97.5%. Under 1, 3, 5, 7 and 10 solar irradiations, the evaporation rates of PPy-wood were 1.33, 3.47, 5.85, 8.38 and 11.77 kg·m^−2^·h^−1^, respectively. They are much higher than the evaporation rates of pure water (0.50, 0.78, 1.19, 1.66 and 2.31 kg·m^−2^·h^−1^) under the same conditions. After treatment with strong acid (pH = 2), strong alkali (pH = 10), high temperature (100 °C) and ultrasound washing, the coating of PPy-wood did not show obvious peeling, which verified its good structural stability. In addition, the multiple scattering of light can be reduced by wood’s rough surface. The light absorption efficiency of PPy-wood is over 93% at various angles (0~60°). Wang et al. [92] prepared PPy-wood by loading the photothermal PPy on balsa wood by in situ polymerization. After one solar irradiation for 1 h, the surface temperatures of water, wood and PPy-wood were 28.2 °C, 32.8 °C and 41.0 °C, respectively. It should be noted that the surface temperature of PPy-wood can reach 39.6 °C within 5 min of illumination. However, the surface temperatures of pure water and wood only increased slightly (Δ_pure water_ ≈ 1.9 °C, Δ_virgin wood_ ≈ 6.5 °C), further confirming that the PPy coating plays an important role in photothermal conversion. The evaporation rate (approximately 1.0 kg·m^−2^·h^−1^) and efficiency (over 70%) hardly changed in seven service cycles. After 45 days of long-term storage, the evaporation rate and efficiency of PPy-wood have no obvious variation.

Qu et al. [93] loaded polyaniline on the surface of natural wood using a spraying method. A low-cost, easily manufactured and high-performance wood evaporator was successfully assembled (Figure 8f). Because of the hydrogen bond between polyaniline and the wood surface, the solar absorption and photothermal conversion efficiency of the evaporator are greatly enhanced. The capillary effect of wood and the hydrophilicity of cellulose facilitate the continuous upward flow of water. Meanwhile, wood’s low thermal conductivity causes the conversion of solar energy to heat at the interface of the wood block. In addition, the porosity and layering of wood blocks decrease the hydrogen bond density of water passing through. Under one solar irradiation, the high temperature of 68.3 °C can be reached within 30 min, verifying the good photothermal conversion effect of a PANI-wood block. In the same condition, the surface temperature of natural wood is relatively stable at 46.2 °C (Figure 8g). The temperature of the evaporator and natural wood tends to be stable after 30 min. This is because the PANI remarkably increases the solar energy absorption of the wood evaporator after modification. The evaporation efficiency of the polyaniline-wood evaporator is 1.66 kg·m^−2^·h^−1^ under one solar irradiation. In addition, the concentration of main ions in purified water obtained by the evaporator fully meets the WHO requirements for drinking water.

Poly (N-phenylglycine) (PNPG) is a conjugated polymer with good light absorption, which can greatly make up for the weak light absorption of wood. In addition, PNPG can effectively improve the photothermal conversion efficiency of wood evaporators. Inspired by the transpiration of trees, Lin et al. [94] developed a PNPG-wood solar evaporator with low thermal conductivity and a special microstructure. Under one solar irradiation, its evaporation rate and conversion efficiency can reach 1.64 kg·m^−2^·h^−1^ and 90.4%, respectively, which are higher than those of most reported wood solar evaporators. This system has the potential to solve the practical issues of seawater desalination and water purification. Li et al. [95] successfully constructed a polyelectrolyte hydrogel (SCPH) with rapid pumping, enhanced salt discharge, and improved mechanical strength and thermal insulation performance. The wood sponge skeleton of SCPH has a series of characteristics such as high mechanical strength, low thermal conductivity, excellent compressibility and high porosity. In addition, the polyelectrolyte hydrogel has weak mechanical strength, high thermal conductivity, and good pumping properties and salt rejection performance. Therefore, a novel SCPH material was developed by making full use of the advantages of polyelectrolyte hydrogel and balsa sponge. The material has a very low thermal conductivity (0.109 W·m^−^^1^·K^−1^), while its mechanical strength is significantly improved. In addition, it is worth noting that its salt rejection rate reached 88.7% even in a 10% salinity solution. The evaporation rate of the SCPH evaporator reached 2.13 kg m^−2^·h^−1^, which greatly exceeded that of a reported polyelectrolyte hydrogel evaporator. It is exciting that the proposed evaporator can run in 20% salt water for 30 days. There is no salt accumulation on its surface, representing an improvement over most reported single-hydrogel evaporators. Sheng et al. [96] used a chemically stable coordination polymer (Ni-DTA) as a hydrophilic photothermal nanomaterial to produce a robust wood-based evaporator (Balsa-NiDTA) with better molecular design performance. Ni-DTA synthesized in situ on the cell wall of balsa wood provides enough light and heat fields, which make the converted energy localize to promote interfacial evaporation. Reasonably controlling the ratio of methanol to dimethylformamide allows 1D nanofibers and 0D nanoparticles to coexist. Therefore, its evaporation rate and energy efficiency under one solar illumination can reach up to 2.75 kg·m^−2^·h^−^^1^ and 82%, respectively. The results show that the Ni-DTA polymer with strong hydration ability will reduce the equivalent evaporation enthalpy, which is attributed to the decrease in the H bond density of water molecules near the evaporation interface. The evaporator shows a high chemical stability, which is mainly attributed to the firm Ni-S/Ni-N bond and the excellent cellulose affinity of Ni-DTA. In addition, the evaporator has excellent antibacterial activity and low oil pollution tendency, which are helpful in realizing the efficient and sustainable solar desalination of Balsa-NiDTA under various harsh conditions.

### 3.5. Wood/Precious Metal Composites

**Figure 9:**
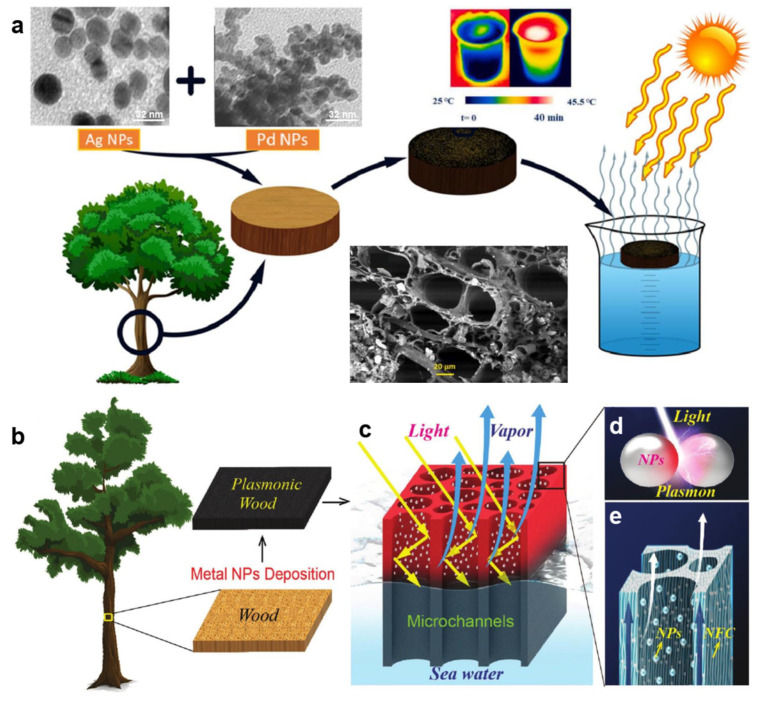
(**a**) Highly efficient plasmonic wood/Ag/Pd photoabsorber in interfacial solar steam generation [104]. Reprinted with permission from Ref. [104]. Copyright 2018 Wiley-Blackwell Ltd. (**b**) Natural wood is cut perpendicular to the growth direction of the tree, and it turns black after nanoparticle decoration due to the plasmonic effect of the metal nanoparticles [105]. (**c**) After metal nanoparticle decoration, light can be guided into the wood lumen and be fully absorbed for steam generation [105]. (**d**) Schematic of plasmonic effect of two adjacent metal nanoparticles (NPs) [105]. (**e**) Zoomed-in schematic illustrating the water transport along microchannels in wood. The cell wall is composed of abundant nanofibrous cellulose (NFC) [105]. Reprinted with permission from Ref. [105]. Copyright 2022 Elsevier Ltd.

When light is incident on a photothermal conversion layer composed of precious metal nanoparticles, if the incident photon frequency matches the total vibration frequency of precious metal nanoparticles or metal conduction electrons, it will have a strong absorption effect on photon energy and produce the local surface plasmon resonance (LSPR) effect [97]. Due to the LSPR effect, precious metal nanoparticles can show a stronger absorption spectrum in the ultraviolet–visible band. In addition, the LSPR effect also causes near-field enhancement, hot electron generation and photothermal conversion [98]. This is because the electrons are excited from an occupied state to an unoccupied state, forming hot electrons [99], the energy of which is redistributed through electron scattering, thus rapidly increasing the temperature of metal particles [100]. The shape and position of LSPR are closely related to the composition, size, shape, dielectric property and dielectric environment of nanoparticles [101]. In general, reducing the shape symmetry or creating a hollow structure can broaden the spectral LSPR band. However, a variation in particle size or the surrounding media will mainly cause a shift in the LSPR band and broaden the absorption band [102]. Silver is widely known for its high plasma resonance effect and low plasma loss. Gold is famous for its visible–near-infrared plasma resonance effect and chemical stability. Therefore, Au NPs and Ag NPs are the most widely used plasmon metals and photothermal materials for solar evaporation [103]. Goharshadi et al. [104] coated layered porous wood with Ag and Pd nanoparticles (NPs) as a double layer. Then, the coated wood could be used as an effective light absorber in interface solar steam generation (ISSG) (Figure 9a). The results showed that the plasmon resonance effect of Ag and Pd NPs could increase the photothermal conversion efficiency of the wood composite when Ag and Pd NPs were used as the bottom layer and upper layer, respectively. The wood composite’s highest evaporation rate was 4.82 kg·m^2^·h^−^^1^ under three solar irradiations. Initially, the salinity, conductivity and pH of seawater were 3138.19 mg·L^−1^, 6000 μS·cm^−^^1^ and 8.1, respectively. After treatment, the salinity, conductivity and pH of seawater were reduced to 6.76 mg·L^−1^, 20.5 μS·cm^−1^ and 6.7, respectively. In addition, the wood composite also presented a stable water generation capability during long-term cycles.

Zhu et al. [105] designed a novel plasmonic wood for efficient steam generation. First, basswood was cut perpendicular to the direction of the tree’s growth. Plasma metal nanoparticles were then deposited in the wood microchannels to form plasma wood (Figure 9b). Black plasmonic wood can float on water without additional assistance. The plasma palladium nanoparticles on the surfaces of microchannels convert incident light into heat due to the plasma effect (Figure 9c,d). Therefore, plasmonic wood has a high solar absorptivity (≈99%) in the wide wavelength range of 200~2500 nm. Due to the thin decorative layer, metal nanoparticles will not block the water microchannels, ensuring continuous water transportation. Due to the low thermal conductivity of plasmonic wood, the heat is concentrated on the evaporation surface, resulting in an effective solar steam generator. The hydrophilicity and capillary effect of plasmonic wood can effectively transport water upward to maintain a continuous water supply for steam generation (Figure 9e). The low thermal conductivity of wood microchannels contributes to thermal localization, which can effectively inhibit heat dissipation to bulk water. Therefore, the plasmonic wood has a unique 3D mesoporous arrangement structure. The solar energy conversion efficiency can reach 85% under 10 solar irradiations. The as-obtained plasmonic wood also showed strong stability (over 144 h) and self-cleaning ability. Thanks to the above advantages, plasmonic wood can be applied to many fields such as water sterilization and seawater desalination.

## 4. Summary and Prospects

Wood has a unique natural pore structure and low thermal conductivity. In addition, it has wide sources, large volume, light weight, good toughness, impact resistance, good biocompatibility, reproducibility and biodegradability. These characteristics lay a solid foundation for its application in water pollution purification and solar-driven seawater desalination. The modification of wood is very beneficial for the full contact and interaction of heavy metal ions, aromatic dyes and bacteria with functional groups and nanomaterials in wood nano/micropores. Apparently, such modified wood composites with a high flux and large adsorption capacity are very suitable for industrial wastewater purification. In addition, wood composites combined with precious metals, semiconductors, polymers and carbon nanomaterials and directly carbonized wood exhibit a unique 3D pore structure, low thermal conductivity, good transpiration characteristics, etc. These merits greatly improve the solar-driven seawater desalination rate and efficiency of wood composites.

### 4.1. Existing Issues

Wood composites are used as photothermal evaporators, filter membranes and adsorbents in the field of water treatment. Many efforts and great breakthroughs have been made in the purification of industrial wastewater containing heavy metal ions, bacteria, aromatic dyes, oil stains, etc. Meanwhile, the catalytic and photothermal properties of nanomaterials and the low thermal conductivity and unique pore structure of wood allow wood composite evaporators to better utilize the full spectrum of sunlight. These outstanding performances accelerate the development of wood composite evaporators in solar-driven seawater desalination. However, there are still many challenges on the road to commercialization; these challenges are listed as follows:

(1) The types of pollutants in actual industrial wastewater are complex. In most reported works, the types of pollutants treated by wood composites are relatively single. The influencing factors and changing rules of industrial wastewater treatment are not investigated deeply enough, and the application scope is still limited.

(2) There are many kinds of woods. Therefore, their pore structures and chemical compositions are quite different. This means that the controllability is relatively low (e.g., uneven distribution of functional groups and nanomaterials and insufficient stability) in the modification of wood (cell walls).

(3) As effective photothermal materials, carbon nanomaterials generally have a high cost, low hydrophilicity, complex production process, etc. These drawbacks cause the uneconomical and poor binding between biomass matrices and carbon nanomaterials.

(4) Although surface carbonization simplifies the preparation of a wood-based interface evaporator, the mechanical strength of carbonized wood is obviously reduced, making structural collapse more likely to occur in harsh environments. In addition, the anisotropic characteristics of wood make it difficult to control its carbonization process and microstructure, which is not conducive to subsequent integration.

(5) Polymers have attracted extensive attention because of their excellent light absorption and mature preparation process. However, the service life and evaporation efficiency of wood/polymer composite solar evaporators are reduced due to the influence of the photodegradation of polymers.

### 4.2. Further Research

Wood composites with excellent performances in the fields of industrial wastewater purification and solar-driven seawater desalination should be further studied in the following aspects:

(1) Multi-directional improvements and multifunctionalization of wood should be enhanced. The 3D nano/micropore structure of wood (conduits or tracheids along the direction of wood growth and wood rays, pits and nanoholes perpendicular to the direction of wood growth) should be made full use of. With reasonable and optimized structure design, suitable nanomaterial loading and functional group modifications on cell walls and surfaces, wood can be endowed with greater value and more functions. This can greatly enrich the types of pollutant treatments and improve the evaporation performance of wood composites with more flexibility in applied environments in wastewater purification and seawater desalination.

(2) Aside from exploring more effective technologies, more simple, feasible and green technical methods should be studied scientifically for fabricating wood composites. The movement path of fluid in wood microchannels should be explored in greater depth. In addition, the effects of hemicellulose/lignin removal technologies and drying methods on wood’s porous structure and its drying shrinkage anisotropy should be studied in depth. The relationship between the hemicellulose and lignin removal and the variation in wood’s porous structure should be investigated carefully. The purpose is to optimize the structure of wood with the appropriate technologies, which should be simple and controllable, green and feasible, low in energy and consumption, and suitable for large-scale production.

(3) The inherent physical and chemical properties of wood have great advantages in the field of polluted water purification and solar-driven seawater desalination, which has built a solid foundation for large-scale and commercial applications. However, research on the application of wood composites in this field is still in the exploratory stage. There is an urgent need to systematically evaluate the multifunctional performance of different woods. The accumulation of data on the practical application and economic effectiveness of wood composites should be enhanced, and a complete development system of wood composites in wastewater purification and seawater desalination should be established.

(4) The advantages and disadvantages of photothermal materials and loading nanomaterials or functional groups should be carefully studied and reasonably combined. The wetting and photothermal properties of a wood composite interface have a great influence on its evaporation performance under solar irradiation. Considering the respective advantages of any raw materials, the development strategy of functional and robust wood composites should be updated and optimized. Wood-based filters and evaporators with low cost, good recombination, high water flux, high photothermal rate, low light reflection, good catalysis, long service life, and simple and feasible characteristics should be prepared more controllably. In addition, it is of great significance to develop wood composites with stimulation response to improve their selectivity and intelligence.

(5) Although the evaporation efficiency of wood composites is high, their solar evaporation is affected by the steam pressure in the limited space. The evaporation result is not ideal in actual seawater desalination applications. Compared with a traditional solar distiller with volume heating, the freshwater productivity of functional wood composites has been improved. However, the service life of wood composites will be reduced in a complex ecological environment with high humidity and high salinity. A similar situation also occurs in the wastewater purification of wood composites. Therefore, future research should not only focus on increasing fresh and clean water production, but also pay more attention to improving wood durability (such as salt resistance, acid and alkali resistance, oil resistance and bacteriostasis).

In summary, there are still many challenges in the fields of industrial wastewater purification and solar-driven seawater desalination. The use of wood composites gives full play to the advantages of renewable resource utilization and effectively solves a series of problems such as the continuous pollution of the ecological environment and the imminent exhaustion of freshwater resources, thus being able to avoid the crisis of water quality and quantity. In addition, it is also a green and sustainable method that provides fresh and clean water for residents’ production and life. Therefore, this review provides a favorable solution for the water shortage issue in remote areas and offers a new idea for alleviating energy consumption and environmental pollution.

## Data Availability

No new data were created or analyzed in this study.
